# Inflammatory rheumatic diseases and the risk of drug use disorders: a register-based cohort study in Sweden

**DOI:** 10.1007/s10067-023-06755-w

**Published:** 2023-08-28

**Authors:** Ali Kiadaliri, Andrea Dell’Isola, Martin Englund

**Affiliations:** 1 Clinical Epidemiology Unit, Department of Clinical Sciences Lund, Orthopedics, Lund University, Lund, Sweden; 2 Clinical Epidemiology Unit, Skåne University Hospital, Remissgatan 4, SE-221 85 Lund, Sweden

**Keywords:** Drug use disorder, Inflammatory rheumatic diseases, Register, Cohort study, Sweden

## Abstract

To investigate the association between chronic inflammatory rheumatic diseases (CIRD) and drug use disorder (DUD). Individuals aged ≥ 30 years in 2009 that met the following conditions were included: residing in the Skåne region, Sweden, with at least one healthcare contact in person and no history of DUD (ICD-10 codes F11-F16, F18-F19) during 1998–2009 (*N* = 649,891). CIRD was defined as the presence of rheumatoid arthritis (RA), ankylosing spondylitis (AS), psoriatic arthritis (PsA), or systemic lupus erythematosus. Treating CIRD as a time-varying exposure, we followed people from January 1, 2010 until a diagnosis of DUD, death, relocation outside the region, or December 31, 2019, whichever occurred first. We used flexible parametric survival models adjusted for attained age, sociodemographic characteristics, and coexisting conditions for data analysis. There were 64 (95% CI 62–66) and 104 (88–123) incident DUD per 100,000 person-years among those without and with CIRD, respectively. CIRD was associated with an increased risk of DUD in age-adjusted analysis (hazard ratio [HR] 1.77, 95% CI 1.49–2.09). Almost identical HR (1.71, 95% CI 1.45–2.03) was estimated after adjustment for sociodemographic characteristics, and it slightly attenuated when coexisting conditions were additionally accounted for (1.47, 95% CI 1.24–1.74). Fully adjusted HRs were 1.49 (1.21–1.85) for RA, 2.00 (1.38–2.90) for AS, and 1.58 (1.16–2.16) for PsA. More stringent definitions of CIRD didn’t alter our findings. CIRD was associated with an increased risk of DUD independent of sociodemographic factors and coexisting conditions.

**Table:** 

**Key Points** • *A register-based cohort study including 649,891 individuals aged≥30 residing in the Skåne region, Sweden, was conducted*. • *Chronic inflammatory rheumatic diseases were associated with higher risks of drug use disorder independent of sociodemographic factors and coexisting conditions*.

**Supplementary Information:**

The online version contains supplementary material available at 10.1007/s10067-023-06755-w.

## Introduction

Chronic inflammatory rheumatic diseases (CIRD), including rheumatoid arthritis (RA), ankylosing spondylitis (AS), and psoriatic arthritis (PsA), are associated with chronic pain, disability, and poor quality of life imposing a considerable burden on people and healthcare systems [1, 2]. Pain relief is thus a high priority in the management of CIRD with analgesics, particularly opioids, increasingly prescribed [3, 4]. However, the evidence supporting the effectiveness of these analgesics for the treatment of chronic non-cancer pain is limited [3, 4].

Indeed, persistent use of analgesics increases the risk of dependence, overdose, addiction and drug use disorders (DUD). For instance, around 10% of people with chronic pain who take opioids on a long-term basis are diagnosed with opioid use disorder [5]. Another study from Canada reported that approximately 1 in 7 individuals with RA had a lifetime diagnosis of substance use disorders [6]. In US, hospitalizations due to DUD including opioids, cannabis, cocaine, and amphetamine rose by 3.5– to 13–fold between 1998–2000 and 2014–2016 in patients with RA [7–9]. However, there is limited epidemiological evidence on risk of DUD from population-based cohorts comparing the risks of DUD among persons with and without CIRD. Moreover, most previous studies were cross-sectional relying on self-reported data. In the present study, we aimed to investigate the associations between CIRD and incident DUD using high-quality register data covering primary and secondary healthcare on the whole population of Skåne, the southernmost region of Sweden.

## Methods

### Data sources

Several registers were used to extract data on the whole population of Skåne, the southernmost region of Sweden, with about 1.4 million inhabitants during 1998–2019: *The Skåne Healthcare Register* for healthcare contacts including publicly practicing physicians' diagnostic codes according to the International Classification of Diseases 10 (ICD-10), *the Longitudinal Integration Database for Health Insurance and Labour Market Studies* for data on education, income, marital status, and immigration status, and t*he Swedish Population Register* for data on sex, age, and place of residence.

### Study design and population

We performed an observational register-based cohort study. Using the Swedish population register, we identified all persons aged 30 years and older residing in Skåne on December 31, 2009 who have been living in the region since January 1, 1998 (*n* = 661,165). To minimize the potential confounding due to propensity to seek care, we excluded 4,712 (0.7%) individuals with no in-person healthcare contact recorded in the Skåne healthcare register. We also excluded 6,562 (1.0%) persons with a DUD diagnosis (as principal or secondary diagnosis) during 1998–2009: opioid use disorder (ICD-10 codes: F11), cannabis use disorder (F12), sedative/hypnotic use disorder (F13), cocaine use disorder (F14), amphetamine use disorder (F15), hallucinogen use disorder (F16), volatile solvents use disorder (F18), and other psychoactive substances use disorder (F19).

### Exposure

Persons with a healthcare contact for CIRD–*the exposure of interest*– as main diagnosis within primary or secondary care in the Skåne Healthcare Register between 1 January 1998 and 30 December 2019 were identified. We used the following ICD-10 codes to identify a CIRD contact: RA (ICD-10 codes: M05–M06, M080, M082), PsA (ICD-10 codes: L405, M070–M073), AS (ICD-10 codes: M45, M081), and systemic lupus erythematosus (SLE) (ICD-10 code: M32). We treated CIRD as a time-varying exposure, that is people who were diagnosed during follow up, we considered them as unexposed over the period prior a CIRD diagnosis and exposed from the diagnosis. Those with a CIRD prior to the start of follow up were considered as exposed for the whole study period.

### Outcomes and follow-up

A first DUD diagnosis (as the main diagnosis) at the Skåne healthcare contact between January 1, 2010 and December 31, 2019 was the *outcome of interest* (incident *DUD).* Each participant was followed from January 1, 2010 until a diagnosis of DUD, death, relocation outside Skåne or December 31, 2019, whichever occurred first.

### Data analysis

We assessed the association between CIRD and incident DUD using the parametric survival models based on restricted cubic splines with 5 degree of freedom to model the baseline hazard and to estimate hazard ratio (HR) with 95% confidence interval (CI) (Stata's “stpm2” command) [10, 11]. We used attained age as the time scale. To assess possible changes in the associations between CIRD and DUD with age (time-varying HR), we estimated models with time-dependent coefficients for CIRD with up to 3 degree of freedom and found that a time-constant coefficient model (proportional hazard) was the preferred model based on the Bayesian information criterion (BIC) and Wald test of time-dependent coefficients (*p*-values were > 0.05).

We conducted our analysis in 3 steps. First, we estimated a model with no adjustment for any covariate (it should be noted that since age was used as the time scale, this model is an *age-adjusted model*). We then estimated a second model adjusted for sex, educational level, marital status, immigration status and income (all measured in the year 2009). Finally, we estimated a model additionally adjusted for Elixhauser comorbidity index [12] as well as anxiety (ICD-10 codes: F40-F41 excluding F412) and personality (ICD-10 code: F60) disorders, measured based on doctor-diagnosed codes in the Skåne healthcare register during 1998–2009 (*fully adjusted model*). In computing Elixhauser comorbidity index, we didn’t count rheumatoid arthritis/collagen vascular diseases. We included anxiety and personality disorders since these are associated with both CIRD and DUD and are not captured by Elixhauser comorbidity index (except ICD-10 code F412) [13, 14].

### Additional analyses

To address the effects of CIRD identification on the estimates, we used two more stringent definitions of CIRD: 1) only contacts registered at least once by a specialist at internal medicine, orthopaedic, or rheumatology clinic; 2) registered on at least two different healthcare contacts with the second contact registered ≥ 30 days after the first contact. We also estimated models for each specific CIRD except for SLE (there were low number of DUD in this subgroup).

## Results

A total of 649,891 individuals were included in the study. Of these, 19,364 (3.1%) had a CIRD diagnosis, of whom 7,755 (40%) were diagnosed during the follow up (i.e. 2010–2019). Compared with the reference cohort, individuals with CIRD were, on average, older, with more comorbidities and lower socioeconomic status and higher proportions of females and Sweden born people (Table 1).

**Table 1:** Baseline characteristics of the sample

	Chronic inflammatory rheumatic diseases	Reference
N	19,364	630,527
Female, n (%)	12,527 (64.7)	326,274 (51.8)
Age, mean (SD)	60 (14.2)	56.8 (15.8)
Elixhauser comorbidity index, mean (SD)	1.4 (1.6)	1.0 (1.4)
Elixhauser comorbidity index, n (%)		
0	7,711 (39.8)	339,587 (53.9)
1	4,968 (25.7)	140,493 (22.3)
2	2,923 (15.1)	70,963 (11.3)
3 and more	3,762 (19.4)	79,484 (12.6)
Level of education, n (%)		
0–9 years of education	5,942 (30.7)	163,452 (25.9)
10–12 years of education	8,792 (45.4)	280,010 (44.4)
≥ 13 years of education	4,549 (23.5)	182,466 (28.9)
Missing	81 (0.4)	4,599 (0.7)
Income quartile, n (%)		
Lowest quartile	5,108 (26.4)	157,694 (25.0)
Quartile 2	5,390 (27.8)	156,960 (24.9)
Quartile 3	4,643 (24.0)	157,629 (25)
Highest quartile	4,223 (21.8)	158,244 (25.1)
Born in Sweden, n (%)	16,274 (84.0)	505,090 (80.1)
Marital status, n (%)		
Never married	3,174 (16.4)	139,039 (22.1)
Previously married	5,419 (28.0)	154,238 (24.5)
Married	10,771 (55.6)	337,250 (53.5)
Anxiety disorders, n (%)	864 (4.5)	24,953 (4.0)
Personality disorders, n (%)	65 (0.3)	2,133 (0.3)

**Fig. 1:**
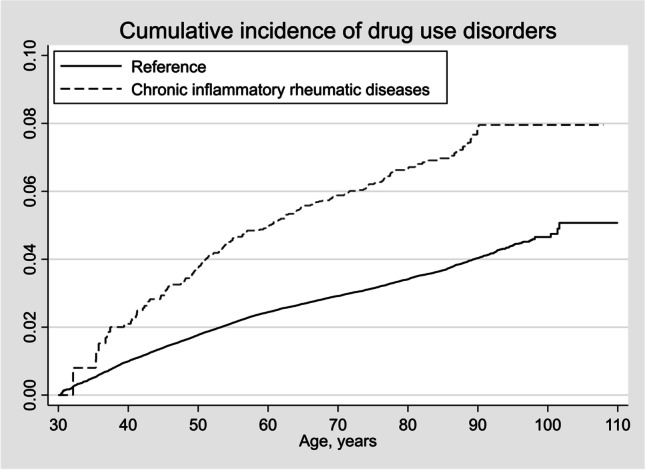
Cumulative incidence of drug use disorder among persons with and without chronic inflammatory rheumatic diseases

During follow up, there were 3,628 and 141 incident DUD in the reference and CIRD cohorts, respectively (Fig. 1), corresponding to 64 (95% CI 62, 66) and 104 (88, 123) incident DUD per 100,000 person-years. The corresponding figures for the different CIRD were: 64 (95% CI 62, 66) vs. 101 (82, 124) for RA, 64 (62, 66) vs. 122 (90, 167) for PsA, and 64 (62, 67) vs. 149 (103, 216) for AS per 100,000 person-years, respectively.

**Fig. 2:**
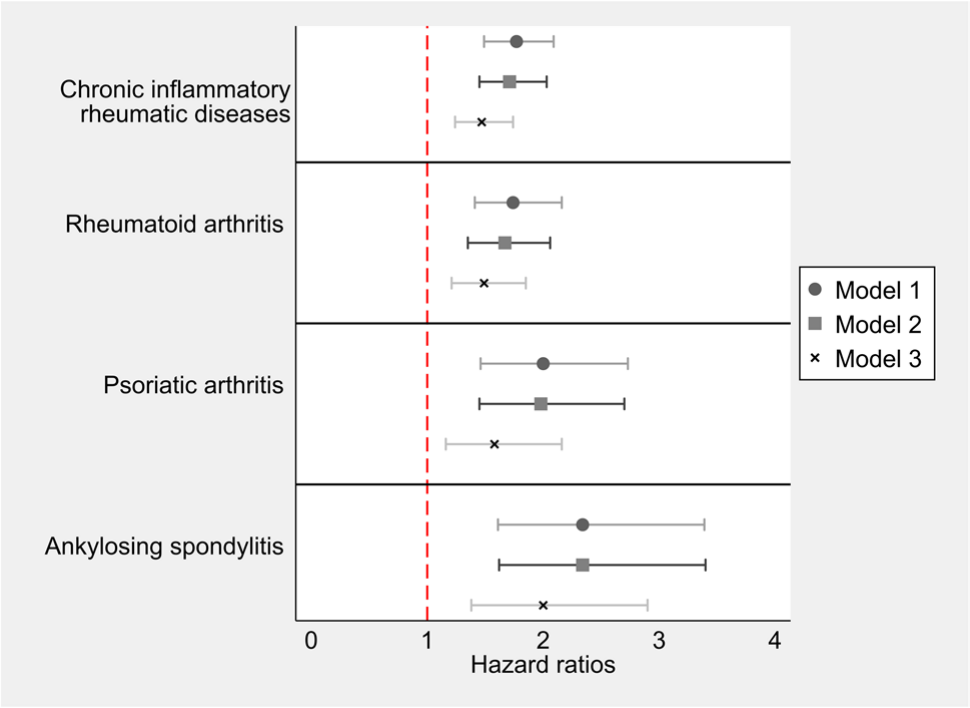
Hazard ratios of drug use disorder associated with chronic inflammatory rheumatic diseases. Model 1: age-adjusted; Model 2: adjusted for age, sex, educational level, marital status, immigration status and income; Model 3: adjusted for age, sex, educational level, marital status, immigration status, income, Elixhauser comorbidity index, anxiety and personality disorders

Our survival analysis suggested that none of the time-dependent coefficients were statistically different from zero and model with time-constant HR had the lowest BIC. Age-adjusted model showed that the DUD rate was 1.77 (95% CI 1.49, 2.09) times higher in CIRD than the reference cohort (Fig. 2). Adjustment for sociodemographic characteristics had almost no impact on this estimate (HR 1.71, 95% CI 1.45, 2.03). However, HR slightly attenuated (1.47, 95% CI 1.24, 1.74) when we additionally adjusted for coexisting conditions. Similar patterns were observed for specific CIRDs with fully-adjusted HRs ranging from 1.49 (95% CI 1.21, 1.85) for RA to 2.00 (1.38, 2.90) for AS. Using more stringent definitions of CRID resulted in essentially similar results (Figs. A1-A2 in appendix).

## Discussion

In this large population register-based cohort study, we found that CIRD was associated with 47% higher rate of incident DUD after adjustment for sociodemographic characteristics and coexisting conditions.

There are mixed results from previous studies that investigated the associations between arthritis and DUD. For instance, while two cross-sectional studies reported higher odds of DUD among persons with arthritis [15, 16], others reported no statistically significant associations [14, 17, 18]. Indeed, McWilliams et al. [14] reported decreased odds of DUD for arthritis which was no longer statistically conclusive after adjustment for age. However, these studies were generally cross-sectional relying on self-reported data on exposure and outcomes which rises the potential for recall and misclassification biases. Moreover, they had smaller sample sizes than the present study and pooled persons with inflammatory and non-inflammatory rheumatic diseases together providing little information on CIRD-specific associations with DUD.

Our results suggested that CIRD is associated with an elevated risk of DUD independently of sociodemographic characteristics and the coexisting conditions. This finding might partially be explained by higher frequency of drug prescriptions in persons with than without CIRD for pain relief which can in turn lead to persistent use and DUD [3]. Moreover, even though we adjusted for Elixhauser comorbidity index at baseline, possibility for residual confounding from coexisting conditions not included in the index should not be overlooked. In addition, there might be differences in developing new comorbidities during follow up between persons with and without CIRD. Chronic pain and substance use disorders are genetically correlated and this shared genetic risk factors might partially explain the elevated risk reported in the present study [19]. While identifying mechanisms underlying increased risk of DUD among persons with CIRD warrant further research, our result call for improvement in drug prescription practices in this population.

Using routinely collected healthcare data covering primary and secondary care with limited selection and no recall bias from a large population-based sample with long follow-up in real-world setting is the main strengths of the present study. However, administrative data sources, like the registers used in this study, are prone to misclassification and coding errors, even though we expect such biases to be less likely for more stringent definitions used in the study. The healthcare register only capture DUD requiring medical care which raise possibility for missing cases with less severity and/or those who don’t seek healthcare. This can bias our estimates if there are differences in proportion of non-identified DUD among persons with and without CIRD. The lack of data on several important confounders such as body mass index, lifestyle factors, family background, pain intensity, disease duration and activity, physical disability, and severity of diseases was another limitation.

## Conclusion

This large population-based cohort study showed higher rates of DUD among persons with than those without CIRD and this couldn’t be explained by sociodemographic factors and coexisting conditions. Our results highlight the need for improvements in pain management and drug prescription practices for persons with CIRD.

### Supplementary Information

Below is the link to the electronic supplementary material.

Supplementary file1 (PDF 485 KB)

## References

[1] Jacobs P, Bissonnette R, Guenther LC (2011). Socioeconomic burden of immune-mediated inflammatory diseases–focusing on work productivity and disability. J Rheumatol Suppl.

[2] Bergman MJ (2006) Social and economic impact of inflammatory arthritis. Postgrad Med 119:5–11 17960689

[3] Anastasiou C, Yazdany J (2022). Review of publications evaluating opioid use in patients with inflammatory rheumatic disease. Curr Opin Rheumatol.

[4] Nowell WB, Gavigan K, Silver SL (2022). Cannabis for rheumatic disease pain: a review of current literature. Curr Rheumatol Rep.

[5] Mallery Lankford C, Paez K, Yang M et al (2022) Adapting the Current Opioid Misuse Measure (COMM) for people with chronic pain and disability due to arthritis: the development of the COMM 11-PWDA. Disabil Health J 15:101296. 10.1016/j.dhjo.2022.101296 10.1016/j.dhjo.2022.101296 35414483

[6] Kowalec K, Carney H, Patel M (2021). Prevalence and risk factors of substance use disorder in rheumatoid arthritis. ACR Open Rheumatol.

[7] Singh JA, Cleveland JD (2021). Time trends in opioid use disorder hospitalizations in gout, rheumatoid arthritis, fibromyalgia, osteoarthritis, and low back pain. J Rheumatol.

[8] Singh JA (2021). Time-trends in cocaine, hallucinogen, amphetamine, and sedative/anxiolytic/hypnotic use disorder hospitalizations in rheumatic diseases: a national time-trends study. Clin Rheumatol.

[9] Singh JA (2021). Comment on: Cannabis use assessment and its impact on pain in rheumatologic diseases: a systematic review and meta-analysis. Rheumatology (Oxford).

[10] Lambert PC, Royston P (2009). Further development of flexible parametric models for survival analysis. Stata Journal.

[11] Royston P, Lambert PC (2011) Flexible Parametric Survival Analysis Using Stata: Beyond the Cox Model. Stata Press, College Station, TX, USA

[12] Quan H, Sundararajan V, Halfon P (2005). Coding algorithms for defining comorbidities in ICD-9-CM and ICD-10 administrative data. Med Care.

[13] Shah R, Zanarini MC (2018). Comorbidity of borderline personality disorder: current status and future directions. Psychiatr Clin North Am.

[14] McWilliams LA, Clara IP, Murphy PD (2008). Associations between arthritis and a broad range of psychiatric disorders: findings from a nationally representative sample. J Pain.

[15] Wu LT, Zhu H, Ghitza UE (2018). Multicomorbidity of chronic diseases and substance use disorders and their association with hospitalization: Results from electronic health records data. Drug Alcohol Depend.

[16] Patten SB, Williams JV, Wang J (2006). Mental disorders in a population sample with musculoskeletal disorders. BMC Musculoskelet Disord.

[17] Stang PE, Brandenburg NA, Lane MC (2006). Mental and physical comorbid conditions and days in role among persons with arthritis. Psychosom Med.

[18] Luther AWM, Reaume SV, Qadeer RA et al (2020) Substance use disorders among youth with chronic physical illness. Addict Behav 110:106517. 10.1016/j.addbeh.2020.106517 10.1016/j.addbeh.2020.106517 32619867

[19] Sanchez-Roige S, Kember RL, Agrawal A (2022) Substance use and common contributors to morbidity: a genetics perspective. EBioMedicine 83:104212. 10.1016/j.ebiom.2022.104212 10.1016/j.ebiom.2022.104212 PMC9399262 35970022

