# Supplementary material for: Inflammatory rheumatic diseases and the risk of drug use disorders: a register-based cohort study in Sweden
Source: Clin Rheumatol. 2023 Aug 28;43(1):81–5. doi: 10.1007/s10067-023-06755-w (PMC10774176; doi:10.1007/s10067-023-06755-w)

Figure A1. Hazard ratios of drug use disorder associated with chronic inflammatory rheumatic diseases (registered at least once by a specialist at internal medicine, orthopaedic, or rheumatology clinic). Model 1: age-adjusted; Model 2: adjusted for age, sex educational level, marital status, immigration status and income; Model 3: adjusted for age, sex educational level, marital status, immigration status, income, Elixhauser comorbidity index, anxiety and personality disorders.

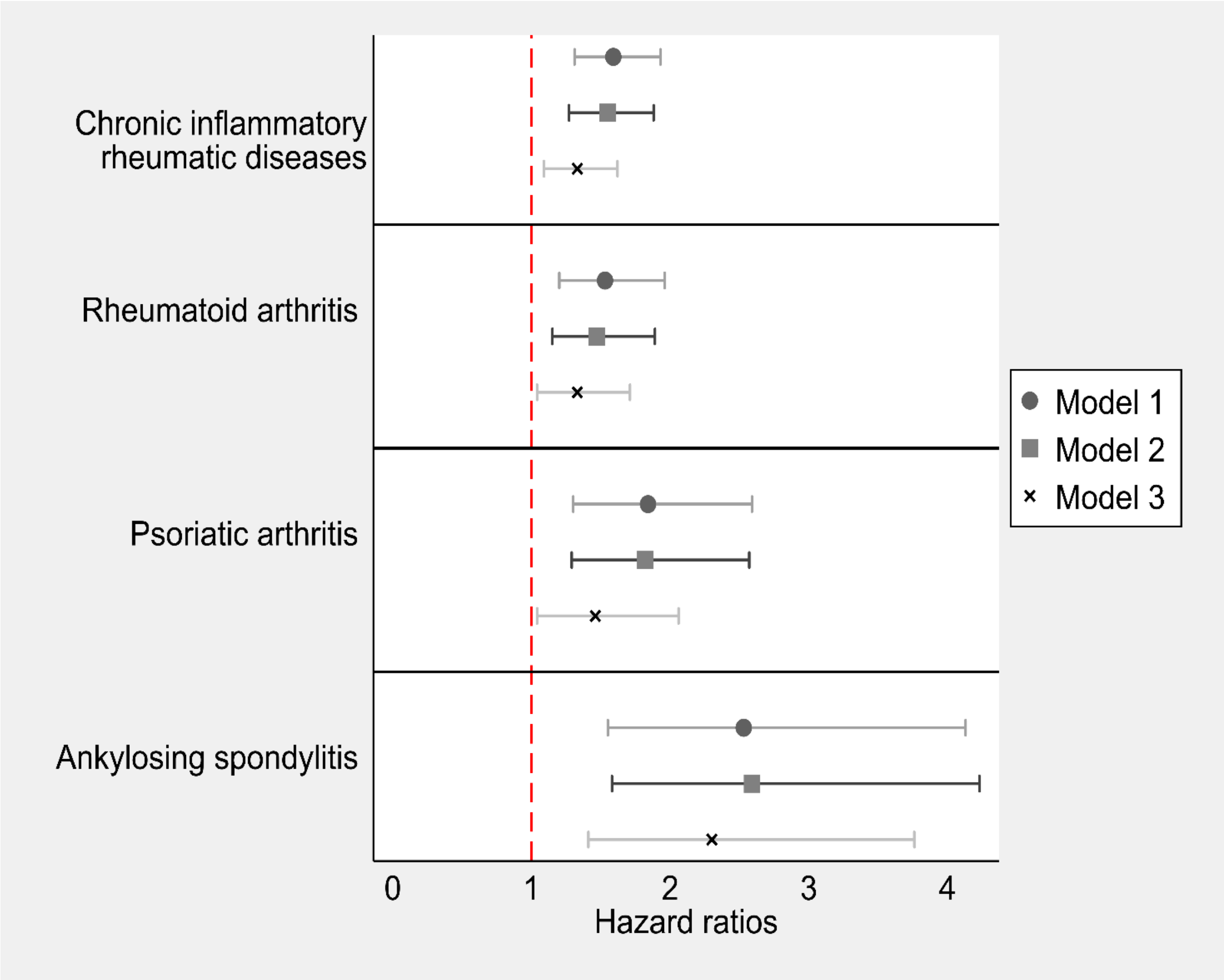

Figure A2. Hazard ratios of drug use disorder associated with chronic inflammatory rheumatic diseases (registered on at least two different healthcare contacts with the second contact registered≥30 days after the first contact). Model 1: age-adjusted; Model 2: adjusted for age, sex educational level, marital status, immigration status and income; Model 3: adjusted for age, sex educational level, marital status, immigration status, income, Elixhauser comorbidity index, anxiety and personality disorders.

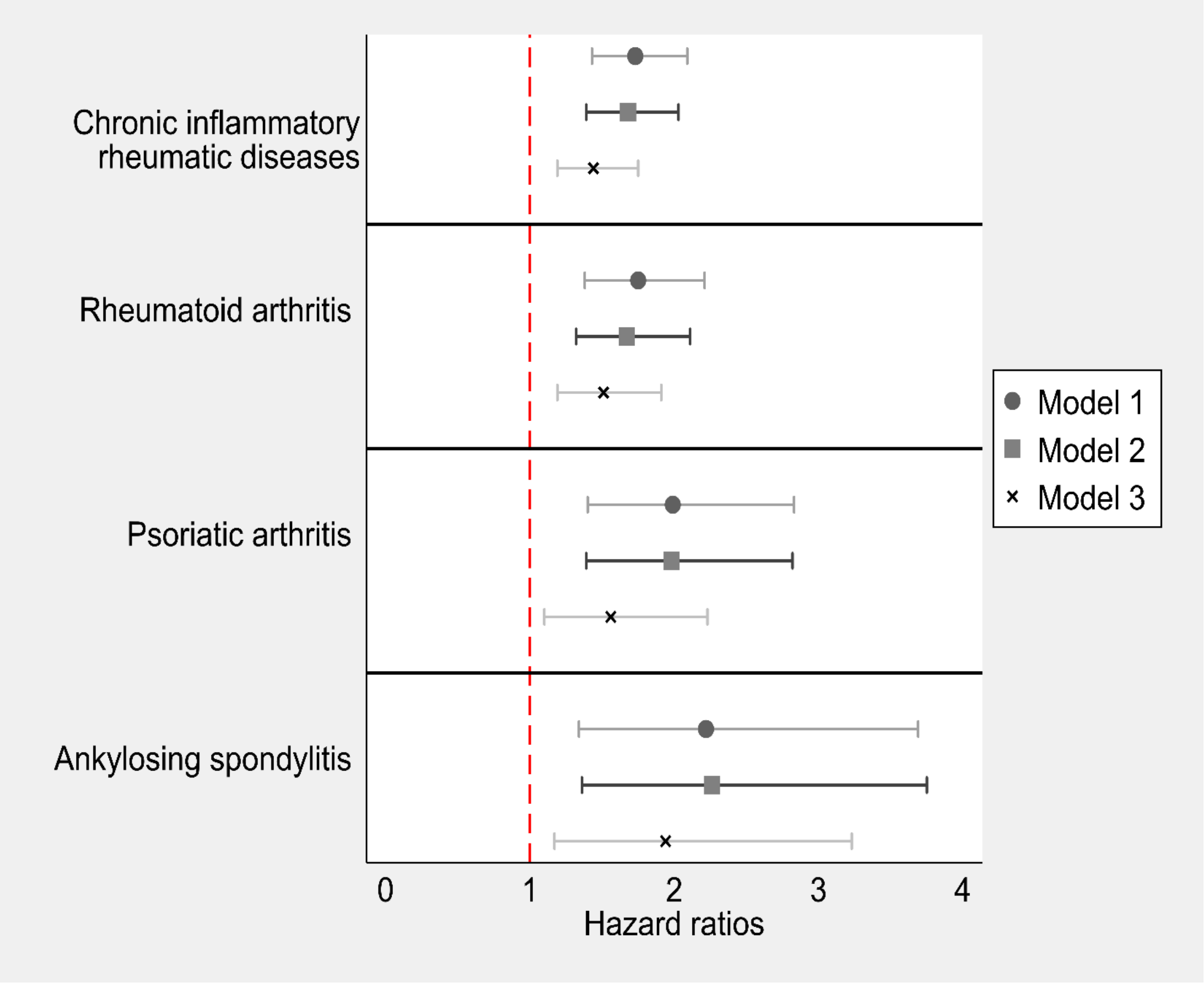

Supplement: Supplementary file 1 — Supplementary file1 (PDF 485 KB) [file 10067_2023_6755_MOESM1_ESM.pdf]
